# Racial differences in long-term adjuvant endocrine therapy adherence and mortality among Medicaid-insured breast cancer patients in Texas: Findings from TCR-Medicaid linked data

**DOI:** 10.1186/s12885-018-5121-z

**Published:** 2018-12-04

**Authors:** Albert J. Farias, Wen-Hsing Wu, Xianglin L. Du

**Affiliations:** 1 Department of Preventive Medicine, Gehr Family Center for Health Systems Science, Keck School of Medicine of the University of Southern California, 2001 N. Soto St., Suite 318B, Los Angeles, CA 90032 USA; 2 Department of Epidemiology, Human Genetics, and Environmental Sciences, School of Public Health, The University of Texas Health Science Center at Houston (UTHealth), Houston, TX USA

**Keywords:** AET, race/ethnicity disparities, adherence, mortality, Medicaid, cancer registry

## Abstract

**Background:**

There are racial/ethnic disparities in breast cancer mortality may be attributed to differences in receipt of adjuvant cancer treatment. Our purpose was to determine whether the mortality disparities could be explained by racial/ethnic differences in long-term adherence to adjuvant endocrine therapy (AET).

**Methods:**

We conducted a retrospective cohort study with the Texas Cancer Registry and Medicaid claims-linked dataset of women (20-64 years) diagnosed with local and regional breast cancer who filled a prescription for AET from 2000-2008. Adherence to AET was measured at three time points (1-, 3-, and 5-year adherence) using a value for the percentage of medication filled for each period divided by the total number of possible prescriptions prescribed (Medication Possession Ratio, MPR). We created a binary variable of adherence (MPR≥80%). We performed multivariable logistic regressions to assess racial differences for the odds of AET adherence and Cox proportional hazard models to determine the risk of mortality adjusting for potential confounding variables of SES, comorbidities, tumor prognostic factors, and other cancer treatment.

**Results:**

Of the 1,497 women with breast cancer who initiated AET, 56.9%, 42.3%, and 33.3% were adherent for 1, 3, and 5-years, respectively. Hispanics compared to non-Hispanic whites did differ in the proportion that were adherent to 5-years of AET. In the adjusted analysis for long-term adherence to AET, Hispanics did not have a significantly increased risk of death compared to non-Hispanic white patients (HR: 1.13, 95% CI: 0.58-2.21). However, black compared to non-Hispanic white patients had significantly lower odds of three-year adherence (OR: 0.45, 95% CI: 0.28-0.73). After controlling for 5-year adherence to AET, the risk of death for black compared to non-Hispanic white patients was 12% lower (HR: 1.90; 95% CI: 1.03-3.51) and in the fully adjusted model, the disparity was reduced and no longer significant (OR: 1.86, 95% CI: 0.94-3.66).

**Conclusions:**

Long-term adherence in the Medicaid population is suboptimal and racial/ethnic differences in AET adherence may partially explain disparities in mortality. This study underscores the critical need to ensure long-term adherence to AET for all racial/ethnic groups to decrease disparities in mortality.

## Background

Minorities with breast cancer have an increased risk of breast cancer death than non-Hispanic whites [1–4]. These racial/ethnic mortality disparities have been attributed to differences in factors such as tumor characteristics [2, 3, 5, 6], socioeconomic factors [4], and the initiation and timing of initial and adjuvant cancer treatment [2, 4]. Adherence to recommended treatment, such as adjuvant endocrine therapy (AET), is one way to significantly reduce breast cancer mortality [7] since adherence to AET is associated with improved disease-free survival for women with early-stage breast cancer [8–12].Thus, racial/ethnic differences in adherence to AET may be a determinant that contributes to disparities in breast cancer outcomes [13].

The National Comprehensive Cancer Network (NCCN) recommends women with hormone receptor-positive breast cancer receive five-years of treatment with either tamoxifen if women are premenopausal or an aromatase inhibitor (exemestane, anastrozole, and letrozole) or tamoxifen if they are postmenopausal [14, 15]. While the benefits of AET are evident, the one-year adherence to recommended treatment (55-75%) is suboptimal [13, 16, 17] and adherence is even lower for racial/ethnic minorities compared white women [18, 19].

Other factors associated with poor adherence include the presence of chronic conditions [20], age at initiation [19, 21], and adverse toxicities of the medication [16, 22–26]. However, despite these associations, medication adherence is often studied within the first year following initiation of therapy. Few studies have examined long-term adherence over the 5-year recommended period, which is important to receive the maximal disease-free survival benefit. Therefore, studying these factors among across the entire 5-year duration of recommended treatment is critical for our understanding of AET adherence.

Moreover, poor adherence to AET is associated low socioeconomic status [18, 27]. In the Texas Medicaid program about one-tenth of low-income adults in the state are covered by the plan and receive comprehensive medical care including prescription drug coverage [29] and enrollment in the program may influence racial/ethnic disparities in cancer mortality in some areas of the state [30–32]. Therefore, we aimed to determine whether racial/ethnic differences in long-term adherence to AET among a low-income and racially diverse Medicaid population explains disparities in all-cause mortality. We hypothesized that AET adherence would be associated with a lower risk of death and, more importantly, there will be no significant disparities in the risk of mortality among Hispanics, Blacks and non-Hispanic whites after controlling for long-term adherence.

## Methods

### Data source and sample population

**Fig. 1:**
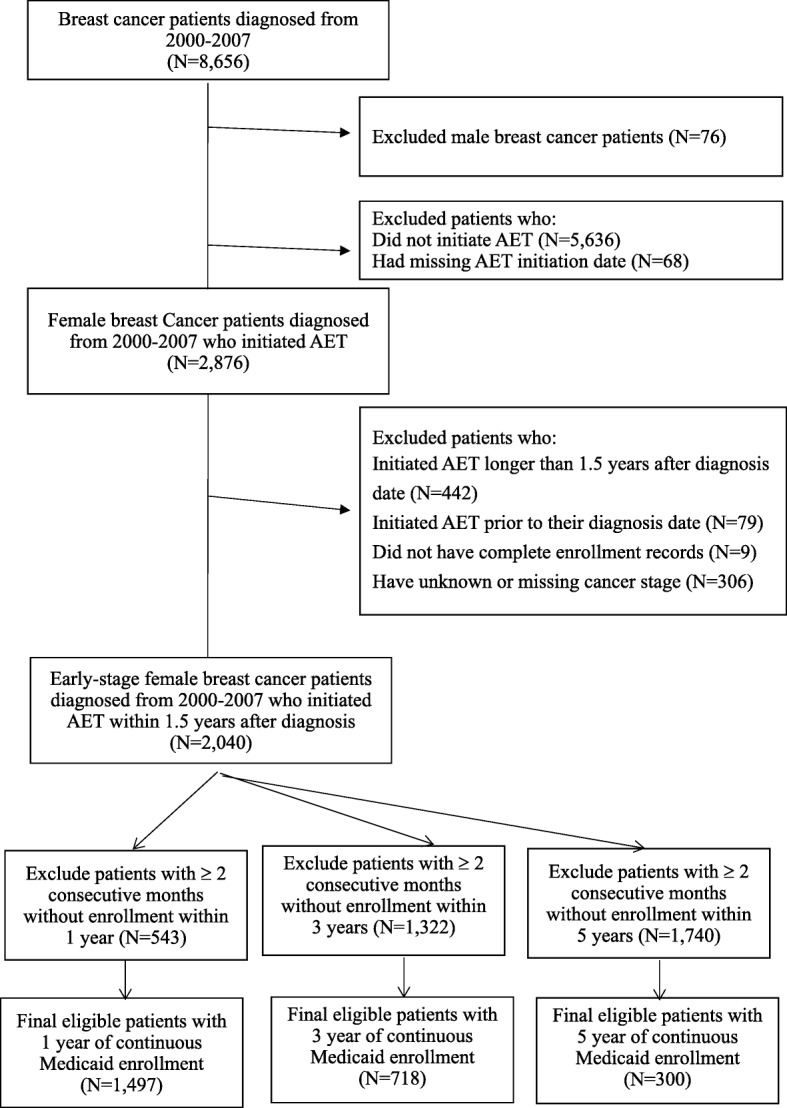
Study diagram

Women with breast cancer aged 20-64 who initiated AET within 1.5 years after the date of cancer diagnosis were identified from Texas Cancer Registry-Medicaid linked database (TCR-Medicaid database) from 2000-2007. Medicaid claims data were available through December 31, 2008 (Fig. 1). The TCR database is a state-based dataset that contains information on initial cancer treatment and clinical and cancer diagnostic characteristics. Data were available for patients enrolled in Medicaid from medical claims for outpatient, inpatient, and pharmacy records.

To calculate long-term adherence, we analyzed three cohorts that had at least one, three, and five years of eligible continuous enrollment in Medicaid after the date of AET initiation. Patients were considered to have eligible continuous enrollment in Medicaid if they did not have two or more consecutive months of unknown or non-enrollment status for each of the adherence time points. Doing this allowed us to accurately assess AET medication prescription refills over each time-period since complete pharmacy claims were available for patients who were continuously enrolled in Medicaid for each time period. Patients may be non-enrolled due to ineligibility in Texas Medicaid, enrolled in another insurance program, or because they moved out of Texas or died. Therefore, these people were excluded from each of the 3 adherence cohorts. However, patients may have been non-enrolled after the 3 time periods or died.

### Outcome Measures

Similar to previous studies, adherence was defined by calculating each patients’ medication possession ratio (MPR) over each of the three time periods (1 year, 3 years, and 5 years) [33–35]. Patients that filled prescriptions for AET to cover more than 80% of the days in one, three, and five years, (MPR≥80%) were defined as adherent. Pharmacy claims from Medicaid include information for the prescription date, days of dugs supplied, and the National Drug Code (NDC). A patient was considered to have initiated AET if they had a pharmacy drug claim identified by the NDC code for either tamoxifen or any of the aromatase inhibitors within 18 months after their diagnosis date. If a patient started taking another AET medication we assumed that they stopped taking any excess prescribed medication for the discontinued AET medication and initiated the new AET drug on the date the new drug was prescribed. We did not count any access AET drug in the medication possession ratio.

All-cause mortality was defined as dead (of any causes). Patients who were alive at the last follow-up of vital statistics (December 31, 2010) were censored. The mean and median follow-up time from breast cancer diagnosis until the end of the study period or death of the one-year adherence cohort in this study were 2,229 days and 580 days (range 438-4,008); of the three-year adherence cohort in this study were 2,678 days and 2,650 days (range 1,114-4,008); of the five-year adherence cohort in this study were 3,188 days and 3,167 days (range 1,918-4,008).

### Race and geographical region variables

We created a single measure for race/ethnicity as identified in Texas Medicaid enrollment file as Non-Hispanic White, Non-Hispanic Black, Hispanic, and other (including Asian or missing race/ethnicity). We included categorical county-level variables for the proportion of the population living below the federal poverty level, median income, and the number of direct primary care (DPC) physicians per 100K population. These variables were categorized into tertiles and based on the 2000 US Census linked to the dataset. Geographic region variables were included and based on the location where patient lived at the time of diagnosis along the Texas-Mexico Border (Yes/No) based on 3-digit county Federal Information Processing Standard (FIPS) codes. These codes were also used to determine whether patients resided in counties that were metropolitan regions or non-metropolitan regions including urban and rural areas based on the US Census Rural-Urban Continuum Codes.

### Demographic and clinical Covariates

Categorical tertiles for age groups and the number of chronic conditions were included as patient characteristics. We measured the number of comorbid conditions between 6 months before and 3 months after breast cancer diagnosis with a modified version of the Charlson comorbidity index (CCI) based on diagnostic and procedures codes described elsewhere [36–38]. Clinical characteristics included tumor stage, receipt of chemotherapy (ICD-9-CM diagnosed code 99.25, V58.1, V66.2, or V67.2, the Common Procedure Terminology codes of 96400-96549, J9000-J9999, or Q0083-Q0085, and NDC drug code 33) [39, 40], radiation therapy (9921-9929, or the CPT codes 77401-77499 or 77750-77799, or revenue center codes 330 or 333) [41], and surgery (ICD-9-CM procedure code 8521-8523, 8541-8548, 19120, 19180, 19222, 19240, 19162) [42]. Our study was limited to patients with localized or regional disease. Year of diagnosis were grouped into three categories: 2000-2001, 2002-2004, 2005-2007.

### Statistical Analysis

Descriptive statistics were used to illustrate patient sociodemographic and clinical treatment and prognostic factors across the racial/ethnic groups. We examined the proportion of adherent patients (MPR≥80%) in the three adherence periods (1, 3 and 5 year) across all variables. We ran three separate multiple logistic regression models to assess the association between race/ethnicity, geographic region, and AET adherence [33–35]. Cox proportional hazard regression was used to analyze the association between adherence to AET at three different time periods and mortality. We conducted six step-wise Cox proportional hazard regression models on each of the three adherence levels (1-, 3-, and 5-year). In the first model, we only included the variable indicating whether a patient was adherent to AET to see the crude risk of mortality associated with adherence at each time period. Next, we added sequentially, 2. race, 3. age, 4. stage and CCI, 5. SES, and 6. initial breast cancer treatment information in order to examine how the association between adherence and risk of death changed with the addition of each variable. Likewise, six regression models were assessed to analyze the impact of race/ethnicity within the three adherence time points in a step wise manner by adding 1. race, 2. adherence, 3. age, 4. stage and CCI, 5. SES, and 6. initial breast cancer treatment information. We checked for multiple collinearity between all variables and used a Pearson’s correlation coefficient r>0.7 or a variance inflation factor (VIF)>7 as a rule of thumb to exclude variables. No variables met these criteria in our final analysis. We used 2-sided p-values and a significance level of p<0.05. We performed all analysis using SAS (version 9.4; SAS Institute, Cary, NC).

## Results

In the first cohort, there were 1,497 breast cancer patients with 1-year of continuous enrollment in the Texas Medicaid after the month of initiating AET. For patients with continuous enrollment for 3 and 5-years there were 718 and 300 patients, respectively. Table 1 lists the characteristics of patients by race. Among 1,497 patients, 566 (37.8%) were Non-Hispanic White, 278 (18.6%) were Non-Hispanic Black and 582 (38.9%) were Hispanic. Minority patients (Hispanic and Blacks) made up over half (57.5%) of our sample population. For patients aged 20-64, 60.3% of them were aged 50-64 years and only 4% of them were younger patients (aged 20 to 34). Approximately 20% of the patients diagnosed with breast cancer resided in a county along the Texas-Mexico border, 44% of all Hispanic breast cancer patients resided in the region compared to only 5.3% and 1.4% as Non-Hispanic White and Black, respectively.

**Table 1:** Characteristics of women with diagnosed with breast cancer who initiated AET within 12 months by race/ethnicity, 2000-2007

	Total		Non-Hispanic White		Non-Hispanic Black		Hispanic		Other		*p*-value
	N	(%)	N	(%)	N	(%)	N	(%)	N	(%)	
Total	1,497	100.0	566	100.0	278	100.0	582	100.0	71	100.0	
Age (years)											<0.001
20-34	60	4.0	13	2.3	13	4.7	33	5.7	1	1.4	
35-49	535	35.7	185	32.7	108	38.9	225	38.7	17	23.9	
50-64	902	60.3	368	65.0	157	56.5	324	55.7	53	74.7	
Tumor stage											0.20
Local	744	49.7	291	51.4	140	50.4	272	46.7	41	57.8	
Regional	753	50.3	275	48.6	138	49.6	310	53.3	30	42.3	
Poverty											<0.001
≤ 16	503	33.6	270	47.7	114	41.0	94	16.2	25	35.2	
16.1-18.3	488	32.6	182	32.2	128	46.0	149	25.6	29	40.9	
≥ 18.4	506	33.8	114	20.1	36	13.0	339	58.3	17	23.9	
Median Income											<0.001
≤ 33,502	479	32.0	122	21.6	34	12.2	309	53.1	14	19.7	
33,503-41946	482	32.2	205	36.2	108	38.9	152	26.1	17	23.9	
≥ 41,947	536	35.8	239	42.2	136	48.9	121	20.8	40	56.3	
DPC Physicians per 100K Population											<0.001
≤103.9	419	28.0	194	34.3	61	21.9	151	26.0	13	18.3	
104-198.7	437	29.2	148	26.2	32	11.5	237	40.7	20	28.2	
≥198.8	641	42.8	224	39.6	185	66.6	194	33.3	38	53.5	
TX-Mexico Border											<0.001
No	1201	80.2	536	94.7	274	98.6	328	56.4	63	88.7	
Yes	296	19.8	30	5.3	4	1.4	254	43.6	8	11.3	
Region											<0.001
Metro	1208	80.7	416	73.5	236	84.9	492	84.5	64	90.1	
Urban/Rural	289	19.3	150	26.5	42	15.1	90	15.5	7	9.9	0.48
Year of Diagnosis											
2000-2001	273	18.2	101	17.8	55	19.8	106	18.2	11	15.5	
2002-2004	659	44.0	258	45.6	130	46.8	240	41.2	31	43.7	
2005-2007	565	37.7	207	36.6	93	33.5	236	40.6	29	40.9	
Chemotherapy											0.20
No	753	50.3	305	53.9	133	47.8	281	48.3	34	47.9	
Yes	744	49.7	261	46.1	145	52.2	301	51.7	37	52.1	
Radiation Therapy											<0.001
No	725	48.4	295	52.1	155	55.8	235	40.4	40	56.3	
Yes	772	51.6	271	47.9	123	44.2	347	59.6	31	43.7	
Surgery											0.22
No	86	5.7	35	6.2	20	7.2	25	4.3	6	8.5	
Yes	1411	94.3	531	93.8	258	92.8	557	95.7	65	91.6	
Comorbidity score											0.06
0	986	65.9	360	63.6	177	63.7	399	68.6	50	70.4	
1-2	333	22.2	144	25.4	58	20.9	121	20.8	10	14.1	
3 or more	178	11.9	62	11.0	43	15.5	62	10.7	11	15.5	

*DPC* direct primary care

One-, three-, and five-year adherence to AET (MPR≥80%) were calculated in this study (Table 2). A greater proportion of patients were adherent to AET at one-year adherence (56.9%) compared to longer-term adherence (three- and five-year), where only 42.3% and 33.3% of patients were adherent to AET. There were certain differences of adherence across the ethnic groups where non-Hispanic Black patients represented the lowest adherence compared to other non-Hispanic whites and Hispanics. For the one-year follow-up cohort, 58.1% and 60.5% of non-Hispanic white and Hispanic patients were adherent to AET. However, only 44.6% of Black patients were adherent. A similar pattern was observed for long-term adherence for the three and five-year cohorts. In the five-year long-term follow-up cohort, only 35.3%, 36.8%, 19.2% were adherent for Non-Hispanic White, Hispanic and Black patients, respectively. A greater proportion of older patients were adherent at three- and five- years compared younger patients.

**Table 2:** Percentage of patient’s adherent to long-term adjuvant endocrine therapy, 2000-2007

	Percent Adherent (MPR≥80%)								
	1-year			3-year			5-year		
	n	%	*p*-value	n	%	*p*-value	n	%	*p*-value
Total Cohort	852	56.9		304	42.3		100	33.3	
Age (years)			<0.01			<0.01			0.18
20-34	23	38.3		1	5.9		0	0.0	
35-49	291	54.4		91	39.6		27	31.4	
50-64	538	59.7		212	45.0		73	35.1	
Race/Ethnicity			<0.001			<0.001			0.12
Non-Hispanic White	329	58.1		127	45.0		43	35.3	
Non-Hispanic Black	124	44.6		33	25.2		10	19.2	
Hispanic	352	60.5		123	46.8		39	36.8	
Other	47	66.2		21	50.0		8	40.0	
Tumor stage			0.10			0.30			0.18
Local	439	59.0		158	44.3		63	37.5	
Regional	413	54.9		146	40.4		37	28.0	
Poverty			<0.01			<0.01			0.15
≤16	269	53.5		118	43.9		48	33.3	
16.1-18.3	262	53.7		77	34.2		16	24.6	
≥18.4	321	63.4		109	48.7		36	39.6	
Median Income			<0.001			<0.05			0.16
≤33,502	309	64.5		121	49.4		42	40.0	
33,503-41946	279	57.9		105	40.4		36	31.6	
≥41,947	264	49.3		78	36.6		22	27.2	
DPC Physicians per 100K Population			<0.05			<0.05			<0.01
≤103.9	252	60.1		84	47.5		31	40.8	
104-198.7	262	60.0		99	45.6		38	40.4	
≥198.8	338	52.7		121	37.4		31	23.9	
TX-Mexico Border			<0.001			<0.05			0.14
No	650	54.1		232	40.1		77	31.4	
Yes	202	68.2		72	51.8		23	41.8	
Region			<0.05			0.46			0.07
Metro	670	55.5		243	41.7		77	31.1	
Urban/Rural	182	63.0		61	45.2		23	44.2	
Year of Diagnosis			<0.05			0.74			0.51
2000-2001	138	50.6		82	44.1		54	35.1	
2002-2004	399	60.6		194	42.2		46	31.5	
2005-2007	315	55.8		28	38.9		0	0.0	<0.05
Chemotherapy			0.09			0.37			
No	445	59.1		171	43.9		67	37.9	
Yes	407	54.7		133	40.6		33	26.8	
Radiation Therapy			<0.05			0.26			0.8
No	392	54.1		145	40.3		52	34.0	
Yes	460	59.6		159	44.4		48	32.7	
Surgery			0.26			0.11			0.38
No	44	51.2		7	26.9		1	16.7	
Yes	808	57.3		297	42.9		99	33.7	
Comorbidity score			0.25			0.86			<0.05
0	569	57.7		186	43.0		48	27.6	
1-2	192	57.7		74	40.7		32	37.2	
3 or more	91	51.1		44	42.7		20	50.0	

*DPC* direct primary care

Table 3 shows the results of the three logistic regression models used to examine the relationship between race/ethnicity and adherence after adjusting for patient characteristics, cancer treatment and regional variables. We found that Black compared to white patients had lower odds of adherence to AET for both one-year adherence (OR: 0.63, 95% CI: 0.47-0.85) and three-year adherence (OR: 0.45, 95% CI: 0.28-0.73). We did not find a significant association between Hispanic and white patients and adherence. In addition, the youngest age group (20-34 years) have significantly lower odds of one-year adherence (OR: 0.46, 95% CI: 0.26-0.82) and three-year adherence (OR: 0.09, 95% CI: 0.01-0.68) compared to the oldest group (50-64 years). Because there were few younger patients in the sample population and younger patients tended not to be adherent, there was no patient in the youngest group which could be followed for the 5-year cohort and thus we did not put age group variable in the third (5-year adherence) model. We also found that breast cancer patients living on the border of Mexico had significantly higher odds of one-year adherence (OR: 2.19, 95% CI: 1.29-3.71) compared to patients not residing in the border counties. Interestingly, patients with three or more comorbid conditions had significantly higher odds of 5-year adherence (OR: 2.87, 95% CI 1.31-6.29), but we did not observe a significant association for one- or three-year adherence.

**Table 3:** Adjusted odds ratio of being adherent to adjuvant endocrine therapy in women with breast cancer, adherence period

	One-year adherence to AET		Three-year adherence to AET		Five-year adherence to AET	
	OR (95% CI)	*p*-value	OR (95% CI)	*p*-value	OR (95% CI)	*p*-value
	*N*=1,497		*N*=718		*N*=300	
Age (years)						
20-34	0.46 (0.26-0.82)	<0.01	0.09 (0.01-0.68)	<0.05	-	-
35-49	0.82 (0.65-1.03)	0.08	0.85 (0.61-1.20)	0.36	-	-
50-64	1		1		-	-
Race/Ethnicity						
Non-Hispanic White	1		1		1	
Non-Hispanic Black	0.63 (0.47-0.85)	<0.01	0.45 (0.28-0.73)	<0.01	0.51 (0.22-1.17)	0.11
Hispanic	0.94 (0.72-1.24)	0.68	0.99 (0.66-1.50)	0.97	1.13 (0.57-2.24)	0.74
Other	1.52 (0.89-2.58)	0.13	1.39 (0.71-2.74)	0.34	1.48 (0.51-4.27)	0.47
Tumor stage						
Local	1		1		1	
Regional	0.86 (0.69-1.08)	0.20	0.93 (0.67-1.30)	0.68	0.56 (0.32-1.00)	0.05
Poverty						
≤16	1		1		1	
16.1-18.3	0.81 (0.59-1.12)	0.20	0.56 (0.35-0.89)	<0.05	0.64 (0.28-1.46)	0.29
≥18.4	0.73 (0.47-1.13)	0.16	0.58 (0.30-1.11)	0.10	0.59 (0.20-1.73)	0.34
Median Income						
≤33,502	1		1		1	
33,503-41946	1.00 (0.65-1.53)	0.99	0.60 (0.32-1.12)	0.11	0.93 (0.35-2.44)	0.88
≥41,947	0.74 (0.44-1.25)	0.26	0.47 (0.22-0.98)	<0.05	0.66 (0.21-2.10)	0.48
DPC^a^ Physicians per 100K Population						
≤103.9	1		1		1	
104-198.7	0.85 (0.62-1.18)	0.34	0.75 (0.46-1.21)	0.24	1.12 (0.51-2.48)	0.78
≥198.8	1.13 (0.80-1.59)	0.50	0.92 (0.55-1.52)	0.74	0.58 (0.25-1.36)	0.21
TX-Mexico Border						
No	1		1		1	
Yes	2.19 (1.29-3.71)	<0.01	1.25 (0.60-2.63)	0.55	1.24 (0.41-3.71)	0.71
Region						
Metro	1		1		1	
Urban/Rural	1.40 (0.95-2.08)	0.09	0.88 (0.50-1.54)	0.66	1.58 (0.62-4.03)	0.34
Year of Diagnosis						
2000-2001	1		1		1	
2002-2004	1.56 (1.15-2.12)	<0.01	1.01 (0.69-1.47)	0.98	0.86 (0.50-1.46)	0.57
2005-2007	1.32 (0.95-1.83)	0.10	0.83 (0.45-1.52)	0.54	-	-
Chemotherapy						
No	1		1		1	
Yes	0.87 (0.69-1.10)	0.24	0.85 (0.61-1.20)	0.36	0.68 (0.39-1.19)	0.18
Radiation Therapy						
No	1		1		1	
Yes	1.27 (1.02-1.58)	<0.05	1.15 (0.83-1.59)	0.41	0.99 (0.58-1.69)	0.98
Surgery						
No	1		1		1	
Yes	1.12 (0.71-1.78)	0.62	2.07 (0.82-5.21)	0.12	1.90 (0.20-18.2)	0.58
Comorbidity score						
0	1		1		1	
1-2	0.98 (0.75-1.28)	0.90	0.83 (0.57-1.20)	0.32	1.51 (0.83-2.76)	0.18
3 or more	0.74 (0.53-1.03)	0.08	0.95 (0.60-1.51)	0.82	2.87 (1.31-6.29)	<0.01

^a^*DPC* direct primary care

We found a significant impact of both short- and long-term adherence on survival (Table 4). In the three study cohorts, approximately 24% of patient died during the follow-up period. Before adjusting for other variables, patients who were adherent to AET had 25-41% lower risk of death compared with non-adherent patients (one-year adherence: 0.75, 0.61-0.92; there-year adherence: 0.60, 0.44-0.83; five-year adherence: 0.59, 0.34-1.01). In the final model, after controlling for patient demographic, treatment and regional variables, the impact of AET adherence on survival became slightly larger for long-term adherence (Five-year adherence: 0.54, 0.30-0.99).

**Table 4:** Cox regression result of the effect of adherence on survival

	One-year adherence to AET		Three-year adherence to AET		Five-year adherence to AET	
Model	HR (95% CI)	*p*-value	HR (95% CI)	*p*-value	HR (95% CI)	*p*-value
Adherence	0.75 (0.61-0.92)	<0.01	0.60 (0.44-0.83)	<0.01	0.59 (0.34-1.01)	0.05
Adherence + Race	0.79 (0.64-0.97)	<0.05	0.64 (0.46-0.89)	<0.01	0.63 (0.36-1.07)	0.09
Adherence + Race+ Age	0.80 (0.65-0.98)	<0.05	0.65 (0.47-0.90)	<0.01	0.64 (0.37-1.10)	0.10
Adherence + Race+ Age+ Stage+ Comorbidity	0.84 (0.68-1.04)	0.12	0.65 (0.46-0.90)	<0.01	0.58 (0.33-1.03)	0.06
Adherence + Race+ Age+ Stage+ Comorbidity+ SES^a^	0.85 (0.68-1.05)	0.13	0.62 (0.44-0.87)	<0.01	0.55 (0.31-0.99)	<0.05
Full model^b^	0.87 (0.70-1.07)	0.19	0.66 (0.47-0.92)	<0.05	0.54 (0.30-0.99)	<0.05

^a^SES variables include Poverty level, DPC physicians per 100 population, TX-Mexico border, Region

^b^Full model includes model 5 plus chemotherapy, radiation therapy, surgery treatment or not and patient year of diagnosis

We also found racial/ethnic disparities in survival among breast cancer patients receiving AET (Table 5). In the unadjusted analysis (model 1) black compared to non-Hispanic white patients had a significantly greater risk of death that ranged from a 36% increased risk of death (HR: 1.36, 95% CI 1.05-1.75) to a two-fold increased risk (HR: 2.02, 95% CI: 1.10-3.71) for those patients with one- and five-year adherence, respectively. However, once we controlled for whether a patient was adherent over the indicated duration, for example 5-years and age (model 3), the risk of death was now 12% lower for black patients compared to non-Hispanic white patients (HR: 1.90, 95% CI: 1.03-3.51). In the fully adjusted model (full model) the racial disparity in mortality was reduced (HR: 1.86, 95% CI: 0.94-3.66) for black compared to non-Hispanic white patients.

**Table 5:** Cox regression model of the effect of socio-demographic characteristics on racial/ethnic disparities in survival

Model	One-year adherence to AET		Three-year adherence to AET		Five-year adherence to AET	
	HR (95% CI)	*p*-value	HR (95% CI)	*p*-value	HR (95% CI)	*p*-value
Model 1: Race						
Black (vs. Non-Hispanic white)	1.36 (1.05-1.75)	<0.05	1.50 (1.02-2.19)	<0.05	2.02 (1.10-3.71)	<0.05
Hispanic (vs. Non-Hispanic white)	0.69 (0.54-0.88)	<0.01	0.81 (0.57-1.17)	0.26	1.15 (0.66-2.02)	0.61
Other (vs. Non-Hispanic white)	0.72 (0.42-1.24)	0.24	0.58 (0.25-1.34)	0.20	0.75 (0.23-2.49)	0.64
Model 2: Race + Adherence						
Black (vs. Non-Hispanic white)	1.30 (1.01-1.69)	<0.05	1.35 (0.92-1.99)	0.13	1.90 (1.03-3.51)	0.04
Hispanic (vs. Non-Hispanic white)	0.69 (0.54-0.89)	<0.01	0.81 (0.56-1.16)	0.25	1.16 (0.66-2.02)	0.61
Other (vs. Non-Hispanic white)	0.73 (0.42-1.26)	0.26	0.59 (0.25-1.35)	0.21	0.77 (0.23-2.55)	0.67
Model 3: Race + Adherence+ Age						
Black (vs. Non-Hispanic white)	1.31 (1.01-1.70)	<0.05	1.34 (0.91-1.98)	0.14	1.85 (0.99-3.46)	0.05
Hispanic (vs. Non-Hispanic white)	0.70 (0.55-0.90)	<0.01	0.80 (0.56-1.15)	0.24	1.16 (0.66-2.04)	0.60
Other (vs. Non-Hispanic white)	0.70 (0.41-1.22)	0.21	0.58 (0.25-1.33)	0.20	0.77 (0.23-2.55)	0.67
Model 4: Race+ Adherence + Age+ Stage+ Comorbidity score						
Black (vs. Non-Hispanic white)	1.20 (0.92-1.56)	0.18	1.17 (0.79-1.74)	0.44	1.61 (0.84-3.10)	0.15
Hispanic (vs. Non-Hispanic white)	0.64 (0.50-0.83)	<0.001	0.69 (0.48-1.00)	0.05	1.01 (0.57-1.79)	0.97
Other (vs. Non-Hispanic white)	0.66 (0.38-1.15)	0.14	0.54 (0.23-1.24)	0.14	0.67 (0.20-2.24)	0.52
Model 5: Race+ Adherence + Age+ Stage+ Comorbidity score +SES^a^						
Black (vs. Non-Hispanic white)	1.24 (0.94-1.62)	0.12	1.27 (0.85-1.91)	0.24	1.75 (0.90-3.40)	0.10
Hispanic (vs. Non-Hispanic white)	0.67 (0.50-0.88)	<0.01	0.70 (0.46-1.08)	0.10	1.10 (0.57-2.11)	0.78
Other (vs. Non-Hispanic white)	0.68 (0.39-1.18)	0.17	0.55 (0.24-1.29)	0.17	0.72 (0.21-2.47)	0.60
Full model^b^						
Black (vs. Non-Hispanic white)	1.23 (0.94-1.62)	0.13	1.29 (0.86-1.93)	0.23	1.86 (0.94-3.66)	0.07
Hispanic (vs. Non-Hispanic white)	0.66 (0.49-0.87)	<0.01	0.72 (0.47-1.12)	0.15	1.13 (0.58-2.21)	0.73
Other (vs. Non-Hispanic white)	0.65 (0.37-1.14)	0.14	0.55 (0.24-1.30)	0.17	0.81 (0.23-2.83)	0.74

^a^SES variables include Poverty level, DPC physicians per 100 population, TX-Mexico border, Region

^b^Full model includes model 5 plus chemotherapy, radiation therapy, surgery treatment or not and patient year of diagnosis

In the unadjusted model (model 1), Hispanic patients had a lower risk of mortality, for the cohort with complete adherence data available for 1-year, compared with Non-Hispanic White patients (HR: 0.69, 0.54-0.88). However, Hispanics did not have significantly decreased risk of death compared to non-Hispanic white patients in the long-term cohorts (5-year cohort; HR: 1.13, 95% CI: 0.58-2.21).

## Discussion

In this study of the Texas Cancer Registry and Medicaid-linked data set, we found that black patients compared to non-Hispanic white patients had significantly lower odds of 1- and 3-year adherence to AET. After adjusting for long-term adherence (5-years), the disparity in mortality between black and non-Hispanic white patients was 12% lower. This is an important finding since in this cohort of patients with comprehensive health coverage and no out-of-pocket costs for medication, adherence for the recommended 5-year period was suboptimal, where nearly two-third of patients did not complete recommended AET treatment.

Prior studies that examined racial/ethnic differences in AET adherence are mixed and vary by insurance status, household income, and out-of-pocket costs for the medication. Studies among privately insured populations did not find significant differences in adherence by race/ethnicity [18, 43, 44], while others that did find a significant racial/ethnic difference in AET adherence are explained by household net worth [18], or out-of-pocket costs for medications [28]. Similar to others, we also found that younger patients had lower odds of adherence to AET treatment within the first year compared to older patients [17, 19].

In our study, merely 33.3% of women were adherent to AET for the five-year recommended duration. Further, we found that 42.3% of patients were adherent to AET for three years. This is lower than previously reported. Partridge and colleagues measured long-term adherence at three years using similar claims-based methodology among non-elderly patients and reported 62–79% were adherent at three years [33]. However, unlike our study, they only had data available for commercially and privately insured patients and did not study adherence among a predominately minority or low-income population, which are associated with poor adherence [33]. Our one-year adherence rate was 56.9% that is similar to other studies using equivalent datasets by population and demographics in North Carolina [20] and New York [45]. These studies found that only three-fifths of patients were adherent at 1-year. Whereas a study using data from the New Jersey Medicaid population included patients with higher incomes found that nearly three-fourths of patients were adherent to AET at one year [19]. Similar to our study, these studies included a racial/ethnically diverse, low-income patient population [20, 45], however, they did not examine long-term adherence by race/ethnicity. Further, we have previously reported AET adherence for stage I-III breast cancer patients enrolled in Medicare Part D and adherence but did not find a significant difference among Hispanic and Black patients over non-Hispanic whites, after controlling for sociodemographic, prognostic and treatment factors [28]. Previous studies have examined lower adherence rates for non-whites, which may explain the disparities in breast cancer mortality observed between minorities and white patients [18, 19, 46]. However, after controlling for AET adherence, we did not observe racial/ethnic differences in the risk of all-cause mortality between black and non-Hispanic white patients. We found that adherence to AET was independently associated with a lower risk of all-cause mortality, which is corroborated by Hershman et al [12]. Similar to our study, they found that Hispanics had a lower risk of death than non-Hispanic whites, [12] but after controlling for long-term adherence we no longer observed this association between Hispanics and non-Hispanic white patients.

In this study, we found that black patients were not statistically more likely to die compared to non-Hispanic white patients after controlling for long-term adherence to AET and age. This is similar to our other finding using the SEER-Medicare dataset among an older publicly insured population [13]. In this study, we found that discontinuing AET treatment was associated with a higher all-cause and cancer-specific mortality (HR: 1.75, 95% CI: 1.54-2.00) [13]. This is important because it demonstrates that adherence to AET, particularly long-term adherence over the recommended 5 years, may reduce the racial/ethnic disparities that we see in cancer outcomes.

While previous studies of AET patterns and outcomes used only medical claims or pharmacy data without details on tumor characteristics [17, 47, 48], we were able to examine long-term adherence and all-cause mortality by other prognostic factors because Medicaid claims data were linked to the TCR registry database. Because we had complete medical claims, pharmacy, and Texas Cancer Registry data, this is one of the most comprehensive studies examining racial/ethnic differences in long-term AET adherence and mortality. To the best of our knowledge, this is the first study to examine adherence to AET among the Texas Medicaid population. Our findings are highly valuable since patients diagnosed with cancer in Texas are not part of the SEER-Cancer Registry and are underrepresented in research studies, which represents a large proportion of minority and low-SES breast cancer patients in the US.

Our study has some limitation. First, our study included women <64 years, continuously enrolled in Texas Medicaid that may have different enrollment eligibility criteria compared to other state-Medicaid programs, and with minority women who may have different life experiences than others throughout the United States. These factors may limit the generalizability of the study findings. Second, unmeasured confounding such as psychosocial factors or factors related to the quality of care women receive (e.g., trust in their physicians) could affect their use of AET but were not measured in this study [49]. Third, our measure of adherence relies on counting the number of prescriptions a patient fills for AET and we assumed that patients were actually taking them. Data were not available in the TCR on estrogen receptor status so we also assumed that patients where eligible to receive AET if they filled a prescription for one of the medications. However, we were able to account for switching AET medication, which allowed us to have a more accurate measure of adherence. Further, using pharmacy claims data has been shown to correlate with medication use and has previously been validated as a method for medication adherence [50, 51]. Finally, we were only able to observe adherence for patients that had continuous follow-up information available for each of the three cohorts. Patients that have continuous enrollment in Medicaid may be different than patients that did not remain enrolled in Medicaid. Because patients that were continuously enrolled in Medicaid did not die during the follow-up time it may make the use of AET seem more effective in this study population. However, patients that were adherent to treatment over the 5-year study period were compared to patients who were alive but did not complete treatment.

## Conclusions

About two-thirds of patients with who initiated AET were non-adherent to AET over the recommended five-year study period. AET adherence was independently associated with a significantly lower risk of all-cause mortality even after adjusting for all other treatment, clinical prognostic, and sociodemographic characteristics. After adjusting to 5-year adherence to AET, we did not observe racial/ethnic differences in all-cause mortality. The findings are novel because they emphasize the importance of improving the low rates of adherence among racial/ethnic minorities as a way to decrease racial/ethnic disparities in cancer mortality.

## Abbreviations

AET: Adjuvant endocrine therapy
CCI: Charlson comorbidity index
CPT: Common procedural terminology
DPC: Direct primary care
HR: Hazards Ratio
ICD-9-CM: International classification of disease, version 9, clinical management
MPR: Medication possession ratio
NDC: National drug code
OR: Odds ratio
SEER: Surveillance Epidemiology and End Results
SES: Socioeconomic status
TCR: Texas Cancer Registry
